# 

**Published:** 1903-01

**Authors:** 


### BOOKS RECEIVED.

A Manual of Dissection and Practical Anatomy. Founded on Gray and Gerrish. By William T. Eckley, M D , Professor of Anatomy in the Medical and Dental Departments of the University of Illinois; the Chicago School of Anatomy and Physiology; the Chicago Clinical School, and West Side Training Schools for Nurses. And Corinne B. Eckley, Demonstrator of Anatomy in the Medical and Dental Departments of the Unive sity of Illinois. Octavo, pp. 414. Illustrated with 220 engravings, 116 of which are colored. Lea Brothers & Co. 1903. [Price, $3.50 net.]

Medical Microscopy. Designed for Students in Laboratory Work and for Practitioners. By T. E. Oertel. M. D., Professor of Histology, Pathology, Bacteriology, and Clinical Microscopy, Medical Department University of Georgia. 12mo, pp. 374. With 131 illustrations, some of which are colored. Philadelphia: P. Blakiston's Son & Co. 1902. [Price, $2.00 net.]

How to Succeed in the Practice of Medicine. By Joseph McDowell Mathews, M. D., LL. D. President Kentucky State Board of Health; Professor of Surgery, Hospital Medical College, Louisville; Author of "Mathews on Diseases of the Rectum." Octavo, pp. 234. Illustrated. Louisville: John P. Morton & Company, 1902. [Price, $2.00.]

The Mattison Method in Morphinism. A Modern and Humane Treatment of the Morphin Disease. By J. B. Mattison, M D., Medical Director, Brooklyn Home for Narcotic Inebriates. 12mo, pp. 40. New York: E. B. Treat & Co. 1902. [Price, $1.00.]

A Reference Handbook of the Medical Science. Embracing the Entire Range of Scientific and Practical Medicine and Allied Science. By various writers. A new edition, completely revised and rewritten. Edited by Albert H. Buck, M. D , New York City. Volume V. Illustrated by chromolithographs and 576 half-tone and wood engravings. Nine volumes, imperial 8vo. New York: William Wood & Co. 1902. [Price, muslin, $6.00 per volume; leather, $7 00 per volume; half morocco, $8.00 per volume.]

Regional Minor Surgery. By George Gray Van Schaick, M. D., Attending Surgeon French Hospital, New York. 226 pages. Profusely illustrated New York: International Journal of Surgery Co. 1902 [Price, $1.50.]

Textbook of Medical Jurisprudence and Toxicology. By John J. Reese, M. D., late Professor of Medical Jurisprudence and Toxicology in the University of Pennsylvania. Sixth edition. Revised by Henry Leffmann, A. M., M. D., Professor of Chemistry and Toxicology in the Woman's Medical College of Pennsylvania. 12mo, pp. 676. Philadelphia: P. Blakiston's Son & Co. 1902. (Price, $3.00 net.]

Transactions of the State Medical Association of Texas. Thirty-fourth Annual Session, held at Dallas, Texas, May 6-9, 1902. H. A. West, M. D., Secretary.

Transactions of the American Otological Society. Thirty-fifth Annual Session, held at New London, Conn., July 16, 1902. Frederick L. Jack, M. D., Secretary. Volume VIII., Part 1. Published by the Society.

